# 2022 IRVING S. WRIGHT AWARD

**DOI:** 10.1093/geroni/igac059.648

**Published:** 2022-12-20

**Authors:** Thomas Gill

**Affiliations:** Yale School of Medicine, New Haven, Connecticut, United States

## Abstract

Dr. Gill is a leading international authority on the epidemiology and prevention of disability among older persons. His nomination for the Wright Award lauded his groundbreaking research on the mechanisms underlying and interventions targeting functional decline and disability among community-living older persons., He collaborates with investigators throughout the country, is a leader of multisite clinical trials, and is a devoted mentor. He has published more than 350 original reports and has been continuously funded by the National Institutes of Health (NIH) and multiple foundations since 1997. Dr. Gill is the Humana Foundation Professor of Medicine (Geriatrics) and Professor of Epidemiology (Chronic Diseases) and of Investigative Medicine; Director, Yale Program on Aging; Director, Claude D. Pepper Older Americans Independence Center; Director, Yale Center for Disability and Disabling Disorders; and Director, Yale Training Program in Geriatric Clinical Epidemiology and Aging-Related Research.

